# Diagnostic yield and risk/benefit analysis of trans-bronchial lung cryobiopsy in diffuse parenchymal lung diseases: a large cohort of 699 patients

**DOI:** 10.1186/s12890-019-0780-3

**Published:** 2019-01-16

**Authors:** Claudia Ravaglia, Athol U. Wells, Sara Tomassetti, Carlo Gurioli, Christian Gurioli, Alessandra Dubini, Alberto Cavazza, Thomas V. Colby, Sara Piciucchi, Silvia Puglisi, Marcello Bosi, Venerino Poletti

**Affiliations:** 1 Department of Thoracic Diseases, G.B. Morgagni - L. Pierantoni Hospital, Via C. Forlanini 34, 47121 Forlì, FC Italy; 2 Interstitial Lung Disease Unit, Royal Brompton Hospital, London, UK; 3 Department of Pathology, G.B. Morgagni - L. Pierantoni Hospital, Forlì, Italy; 4 Department of Pathology, S. Maria Nuova Hospital-I.R.C.C.S, Reggio Emilia, Italy; 5 Department of Pathology, Mayo Clinic, Scottsdale, AZ USA; 6 Department of Radiology, G.B. Morgagni - L. Pierantoni Hospital, Forlì, Italy; 7 Department of Respiratory Diseases and Allergy, Aarhus University Hospital, Aarhus, Denmark

**Keywords:** Cryobiopsy, Transbronchial lung cryobiopsy, TLCB, ILD, Interstitial lung disease, DPLDs, Diffuse parenchymal lung disease, IPF, Idiopathic pulmonary fibrosis

## Abstract

**Background:**

Standardization of trans-bronchial lung cryobiopsy in diffuse parenchymal lung diseases is imminent; however, the majority of published series on cryobiopsy include a limited number of patients and are characterized by several differences in procedural technical details.

**Methods:**

This is an observational, retrospective cohort study. Aim of the study was to suggest some sampling strategies related to transbronchial cryobiopsy in the diagnostic work-up of patients with diffuse parenchymal lung diseases.

**Results:**

Six hundred ninety-nine patients with suspected diffuse parenchymal lung disease were recruited. A specific pathological diagnosis was achieved in 614/699 cases (87.8%) and a multidisciplinary diagnosis was obtained in 630/699 cases (90.1%). Diagnostic yield was significantly influenced by the number of samples taken (1 vs ≥ 2 biopsies, *p* < 0.005). In 60.4% of patients, biopsies were taken from one site and in 39.6% from different sites (in the same lobe or in two different lobes), with a significant increase in diagnostic yield, specifically in patients with fibrotic lung diseases (65.5% vs 93.4%, *p* < 0.0001). The 2.4 mm or 1.9 mm probes were used, with no differences in terms of diagnostic yield. Regarding safety, pneumothorax occurred in 19.2% and was influenced by baseline lung function; in all patients Fogarty balloon has been used and severe haemorrhage occurred in 0.7% of cases. Three patients (0.4% of cases) died within 30 days after the procedure.

**Conclusions:**

We propose some sampling strategies of cryobiopsy which seem to be associated with a higher diagnostic yield and a favorable risk/benefit ratio: sampling at least two samples in different sites, using either the 2.4 mm or the 1.9 mm probe, intubating the patients and using bronchial blockers/catheters.

## Background

While standardization of the cryobiopsy in the diagnostic process of diffuse parenchymal lung diseases is imminent, series reporting experience of cryobiopsy generally include limited number of patients. It is difficult to compare series due to differences in sampling strategies and procedural technical details (such as the use of bronchial blockers to minimize haemorrhage), resulting in major differences in the diagnostic yield and prevalence of complications. We report the largest series of patients with suspected diffuse parenchymal lung disease undergoing transbronchial lung cryobiopsy and we propose a sampling strategy which is associated with a high diagnostic yield and a favorable risk/benefit ratio.

## Methods

We identified from our database all subjects who had undergone trans-bronchial lung cryobiopsy (TLCB) at the Pulmonology Unit of G.B. Morgagni – L. Pierantoni Hospital in Forlì (Italy) for diagnosis of diffuse parenchymal lung diseases from March 2011 through September 2017. All subjects had suspected diffuse parenchymal lung diseases with non-diagnostic clinical profiles, CT scan features (either fibrotic or non-fibrotic), and laboratory tests (including autoimmune serology and precipitins) for whom a biopsy was deemed useful for a diagnosis were prospectively enrolled.

Bronchoscopies were performed as previously described [1]: a 1.9 mm or 2.4 mm cryoprobe was used (ERBE, Germany) and patients were deeply sedated (using propofol and remifentanil), maintained in spontaneous breathing and intubated with a rigid tracheoscope. Biopsies were obtained under fluoroscopic guidance at a distance of approximately 10 mm from the thoracic wall. Bronchoscopic cryobiopsy was targeted to the areas of abnormality seen on HRCT (high resolution computed tomography), with samples taken from one site or multiple sites depending on the radiological pattern and distribution of disease; in particular, cryobiopsy was performed in different sites in patients with significant radiographic inter-lobar heterogeneity, while in patients with diffuse radiographic pattern (both in the upper and the lower lobes) or in patients with a significant apical-basal gradient cryobiopsy was more frequently performed in the same lobe. The choice of the site and side of biopsy was decided upon before the procedure. Biopsies obtained from the middle lobe and the lingula were included in the analysis and compared for both diagnostic yield and complications (being excluded only in the specific analysis evaluating the differences between upper lobes and lower lobes). The probe was cooled for approximately 5–6 s or 7–8 s for the 2.4 mm and 1.9 mm diameter respectively. The frozen specimens were thawed in saline and then transferred gently to formalin for fixation. A Fogarty balloon was always routinely used to prevent severe bleeding. As previously described [2], bleeding was defined as “mild” if requiring just endoscopic aspiration, “moderate” if requiring further endoscopic procedures (bronchial occlusion and/or instillation of ice-cold saline), and “severe” if requiring surgical interventions, transfusions and/or admission to intensive care unit for hemodynamic or respiratory instability. Within 3 hours of the procedure, a chest radiograph was performed to assess for pneumothorax.

In the first 310 of this series, specimens were reviewed by three expert lung pathologists (AD, AC and TVC); the remaining cases were reviewed by AD and AC and only in case of discordancy, a consensus diagnosis was reached after consultation with a third pathologist (TVC).

Biopsies were considered “non diagnostic” when histopathologic criteria sufficient to define a characteristic histopathologic pattern were lacking (eg. normal lung or minimal nonspecific changes) or when samples were considered inadequate (eg, too small or airway wall with no alveolated lung parenchyma). Clinical information, radiological features and biopsy results were then reviewed by clinicians, radiologist and pathologists and a multidisciplinary diagnosis was made, with cryobiopsy considered diagnostic if additional evaluation, including surgical lung biopsy, was considered to be unnecessary.

### Statistical analysis

Statistical analyses were performed using Fisher exact test, Mann-Whitney U test and univariate/multivariate Cox regression analyses; SPSS statistics and STATA (version 12, StatCorp, College Station, TX, USA) were used. A *p* value of < 0.05 was considered statistically significant.

## Results

During the study period, 699 subjects with a median age of 61 ± 11 years underwent cryobiopsy for evaluation of diffuse parenchymal lung disease. Some of these patients have been included in other published series relating to transbronchial lung cryobiopsy [1–4]. Subject characteristics are summarized in Table 1. In 422 patients (60.4%), biopsies were taken from one site, in 267 patients (38.2%) from two sites and in 10 cases (1.4%) from three different sites. Different sites were represented by different segments of the same lobe (in 166 cases) or segments of two different lobes (in 101 cases). Average number of fragments was 3.3 (range 1–11). 2.4 mm probe has been used in 613 patients and 1.9 mm probe has been used in 73 patients (in cases when excessive resistance during retrieval of the 2.4 mm probe was observed due to bronchomalacia or when sampling in the upper lobes with the 2.4 mm probe was particularly difficult). Pleural tissue was detected in 177 cases (25.3%). Mean surface of samples was 30, 35 mm^2^ +/− 18,4 (range 1,51 – 392,4). Biopsy characteristics are summarized in Table 2.

**Table 1:** Clinical characteristics, diagnostic yield and complications in patients submitted to trans-bronchial lung cryobiopsy (TLCB)

Patient characteristic (tot 699)	No. (% or SD)
Median age (SD), y	61 (11)
Male, No. (%)	413 (59.1%)
Mean FVC percent predicted (SD)	85.4 (19.7)
Mean DLCO percent predicted (SD)	61.2 (17.5)
Pathological diagnosis, No. (%)	614 (87.8)
Multidisciplinary diagnosis, No. (%)	630 (90.1)
Pneumothorax, No. (%)	134 (19.2)
Drained Pneumothorax (among those with pneumothorax), No. (%)	94 (70.1)
Mild bleeding, No. (%)	29 (4.1)
Moderate bleeding, No. (%)	53 (7.6)
Severe bleeding, No. (%)	5 (0.7%)

Abbreviations: *FVC* Forced vital capacity, *DLCO* Diffusing capacity of the lungs for carbon monoxide

**Table 2:** Biopsy characteristics and sampling strategy in patients submitted to trans-bronchial lung cryobiopsy (TLCB)

Biopsy characteristics	No. (% or range)
Number of samples	3.3 (1–11)
Probe size	
- 2,4 mm	- 613 (87.7%)
- 1,9 mm	- 73 (10.4%)
- 2,4 + 1,9 mm - unknown	- 11 (1.6%) - 2 (0.3%)
Sample site	
- One site	- 422 (60.4%)
- Two different sites	- 267 (38.2%)
	- 166 same lobe - 101 different lobes
- Three different sites	- 10 (1.4%)
Cryobiopsy smallest axis diameter	4.57 mm +/− 1.18 (0.86–9.81 mm)
Cryobiopsy largest axis diameter	6.31 +/− 1.91 mm (range 1.61–20.12).
Pleural tissue present (visceral and/or parietal)	177 (25.3%)
Sample surface area	30.35 mm2 +/− 18.4 (range 1,51–392.4)

A specific pathological diagnosis was achieved in 614/699 cases (87.8%). The pathologic interpretations are shown in Table 3, including 262 (37.5%) UIP (usual interstitial pneumonia), 66 (9.4%) NSIP (non-specific interstitial pneumonia) or OP/NSIP (organizing pneumonia/non-specific interstitial pneumonia), 58 (8.3%) OP (organizing pneumonia), 36 (5.2%) DIP/RB-ILD (desquamative interstitial pneumonia/respiratory bronchiolitis-interstitial lung disease), 47 (6.7%) malignancy, 38 (5.4%) sarcoidosis, 33 (4.7%) HP (hypersensitivity pneumonitis) and 21 bronchiolitis (3.0%). Among patients with UIP pattern on biopsy, in 58% of cases the pathological diagnosis of UIP was done with high level of confidence (patchy fibrosis and fibroblastic foci with or without honey-combing and no ancillary findings against IPF). When histology has been reviewed by three pathologists, the overall interpersonal agreement between pathologists for the diagnosis of UIP pattern was 0.72 (0.64–0.80) and the overall agreement for the level of confidence in the diagnosis of UIP pattern was 0.54 (0.45–0.62).

**Table 3:** Histopathologic pictures of patients undergoing trans-bronchial lung cryobiopsy

Morphological diagnosis	No. (%)
UIP pattern	262 (37.5%) - 236 UIP - 7 UIP + PPFE - 19 UIP-HP
HP pattern	33 (4.7%) - 31 HP - 2 HP + PPFE
OP	58 (8.3%)
NSIP	43 (6.2%)
OP/NSIP	23 (3.3%)
Malignancy	47 (6.7%)
	- 33 epithelial neoplasms - 14 lymphoproliferative disorders
Sarcoidosis	38 (5.4%)
DIP/RB-ILD	36 (5.2%)
Follicular/constrictive bronchiolitis	24 (3.4%)
LCH	7 (1.0%)
Pneumonia	7 (1.0%)
Aspiration pneumonia/lipoid pneumonia	7 (1.0%)
Silicosis	6 (0.9%)
PPFE	5 (0.7%)
Other	18 (2.6%) - 4 DAD - 4 AFOP - 2 GL-ILD ^a^ - 2 Lymphoid Nodular Hyperplasia - 2 alveolar proteinosis - 1 DIPNECH - 1 ACFE - 1 ECD - 1 vasculitis
ND	85 (12.2%)

Abbreviations: *UIP* Usual interstitial pneumonia, *PPFE* Pleuro-parenchymal fibroelastosis, *HP* Hypersensitivity pneumonitis, *OP* Organizing pneumonia, *NSIP* Non-specific interstitial pneumonia, *DIP* Desquamative interstitial pneumonia, *RB-ILD* Respiratory bronchiolitis-interstitial lung disease, *LCH* Langerhans cell histiocytosis, *DAD* Diffuse alveolar damage, *AFOP* Acute fibrinous organizing pneumonia, *GL-ILD* Granulomatous lymphocytic-interstitial lung disease, *DIPNECH* Diffuse idiopathic pulmonary neuroendocrine cell hyperplasia, *ACFE* Airway-centered fibroelastosis, *ECD* Erdheim Chester disease.

^a^Diagnosis based on clinicopathologic correlation

A multidisciplinary diagnosis was possible in 630/699 cases (90.1%). The most common diagnosis (245/699) was UIP/IPF (idiopathic pulmonary fibrosis). Other diagnoses are displayed in Table 4. In the remaining 20 patients (2.9%), disease was considered to be unclassifiable. Among the 69 subjects with non-diagnostic or uncertain cryobiopsies, 4 patients repeated cryobiopsy (final diagnoses were 1 alveolar proteinosis, 1 IPF, 1 lymphoma and 1 confirmed ACFE = airway centered fibroelastosis), 38 patients (5.4%) underwent surgical lung biopsy (diagnoses were 1 COP = cryptogenic OP, 16 IPF, 1 vasculitis, 1 cocaine-lung, 3 chronic HP, 1 confirmed ACFE, 1 confirmed ECD = Erdheim Chester disease, 4 diffuse lung cancer, 3 iNSIP, 2 RB-ILD, 1 lymphoma, 1 Langerhans cell Hystiocytosis, 1 alveolar proteinosis, 1 CTD-ILD = connective tissue disease related ILD, 1 diffuse inflammatory myofibroblastic tumour), 6 patients underwent CT-guided percutaneous lung biopsy (diagnoses were 3 diffuse lung cancer, 1 lymphoma, 1 COP, 1 not diagnostic) and 1 patient underwent surgical mediastinoscopy (sarcoidosis).

**Table 4:** Final multidisciplinary diagnoses in patients undergoing trans-bronchial lung cryobiopsy (TLCB)

Multidisciplinary diagnosis	No. (%)
IPF	245 (35.1%) - 229 sporadic IPF - 16 familial IPF
CTD	50 (7.2%)
COP	47 (6.7%)
HP	45 (6.4%)
Sarcoidosis	39 (5.6%)
DIP/RB-ILD	36 (5.2%)
Malignancy	48 (6.9%) - 33 epithelial neoplasms - 15 lymphoproliferative disorders
iNSIP	28 (4.0%)
Follicular/Constrictive bronchiolitis	17 (2.4%)
Pneumonia	16 (2.3%)
DR-ILD	9 (1.3%)
Aspiration/lipid pneumonia	7 (1.0%)
LCH	7 (1.0%)
Silicosis	6 (0.9%)
iPPFE	6 (0.9%)
CEP	5 (0.7%)
AFOP	4 (0.6%)
Other	15 (2.1%) - 3 GVHD - 2 lymphoid nodular hyperplasia in CVID - 2 GL-ILD - 2 alveolar proteinosis - 1 DAD - 1 DIPNECH - 1 ACFE - 1 ECD - 1 vasculitis - 1 asthma
ND	69 (9.9%)

Abbreviations: *IPF* Idiopathic pulmonary fibrosis, *CTD* Connective tissue disease, *COP* Cryptogenic organizing pneumonia, *HP* Hypersensitivity pneumonitis, *RB-ILD* Respiratory bronchiolitis-interstitial lung disease, *iNSIP* Idiopathic, *NSIP* Non-specific interstitial pneumonia, *DR-ILD* Drug-related interstitial lung disease, *LCH* Langerhans cell histiocytosis, *iPPFE* Idiopathic pleuro-parenchymal fibroelastosis, *CEP* Chronic eosinophilic pneumonia, *AFOP* Acute fibrinous organizing pneumonia, *GVHD* Graft versus host disease, *CVID* Common variable immune-deficiency, *GL-ILD* Granulomatous lymphocytic-interstitial lung disease, *DAD* Diffuse alveolar damage, *ACFE* Airway-centered fibroelastosis, *ECD* Erdheim Chester disease

Yields for both pathological and final multidisciplinary diagnoses were influenced by the number of samples taken. After a single biopsy, the diagnostic yield was 67.6%, rising strikingly with a second biopsy to 91 and 87% for pathological and multidisciplinary diagnosis respectively. The diagnostic yields did not increase further if more than two samples were taken (Table 5). The diagnostic yield was also influenced by the sampling strategy: yields of both pathological and multidisciplinary diagnoses were significantly increased when biopsies were taken from two sites instead of only one site (247/267, 92.5% vs 358/422, 84.8%, *p* = 0,001 and 248/267, 92.9% vs 373/422, 88.4%, p = 0,043 respectively) (Table 6), although yields did not differ whether sites were represented by different segments of the same lobe (eg posterior and lateral segment of the right lower lobe) or segments coming from different lobes (eg. right lower lobe and right upper lobe) (Table 6). Specifically, considering only patients with fibrotic lung diseases undergoing cryobiopsy in two sites (*n* = 197), the diagnostic yield from a single site was 65.5%, increasing to 93.4% with sampling from a second site (*p* < 0.0001). Samples from two sites were considered concordant if they showed the same pattern (eg, UIP pattern in the lower lobe and UIP pattern in the upper lobe) and discordant if they showed different patterns (eg, NSIP in the upper lobes and UIP in the lower lobes); cases in which the biopsy was inadequate or non-diagnostic in both sites were excluded. Discordant samples between the two sites were observed in 55 patients (27.9%). As shown in Table 6, diagnostic yield did not differ between the 1.9 mm and 2.4 mm probes. These results were confirmed following correction for sampling strategy and number of samples taken (probe size: odds ratio 1.48, *p*-value = 0.277, CI 95.0% 0.73–3.00; samples number: odds ratio 3.65, p-value = 0.001, CI 95.0% 1.65–8.07; sampling strategy: odds ratio 1.82, p-value = 0.046, CI 95.0% 1.01–3.26).

**Table 5:** Correlation between safety outcome and diagnostic yield with number of samples

	1–2 samples			≥ 3 samples	Fisher’s exact test
Pneumothorax	19/166 (11.4%)			115/532 (21.6%)	p 0,0009
Pathological diagnosis	145/168 (86.3%)			469/531 (88.3%)	p 0,5030
	1 sample	2 samples	p		
	23/34 (67.6%)	122/134 (91.0%)	0,0090		
Multidisciplinary diagnosis	20/168 (11.9%)			49/531 (9.2%)	p 0,3406
	1 sample	2 samples	p		
	23/34 (67.6%)	125/134 (87.0%)	0,0042

**Table 6:** Differences in terms of safety outcome and diagnostic yield between different sampling strategies

	1 site	2 sites	Fisher’s exact test
Pneumothorax	64/420 (15.2%)	66/268 (24.6%)	p 0,002
Bleeding	51/418 (12.2%)	32/266 (12.0%)	p 0,947
Pathological diagnosis	358/422 (84.8%)	247/267 (92.5%)	p 0,001
Multidisciplinary diagnosis	373/422 (88.4%)	248/267 (92.9%)	p 0,043
	1 lobe	Different lobes	Fisher’s exact test
Pneumothorax	36/166 (21.7%)	30/102 (29.4%)	p 0,083
Bleeding	19/166 (11.4%)	13/100 (13%)	p 0,7112
Pathological diagnosis	155/166 (93.4%)	92/101 (91.1%)	p 0,5081
Multidisciplinary diagnosis	156/166 (93.9%)	92/101 (91.1%)	p 0,3967
	Upper lobes (^a^)	Lower lobes (^a^)	Fisher’s exact test
Pneumothorax	4/80 (5%)	57/298 (19.1%)	p 0,00004
Bleeding	5/78 (6.4%)	42/298 (14.1%)	p 0,0270
	1.9 probe	2.4 probe	Fisher’s exact test
Pneumothorax	2/73 (2.7%)	130/613 (21.2%)	p < 0,0001
Bleeding	8/73 (10.9%)	78/611 (12.8%)	p 0,6460
Pathological diagnosis	62/73 (84.9%)	541/615 (87.9%)	p 0,4936
Multidisciplinary diagnosis	62/63 (84.9%)	557/615 (90.6%)	p 0,2014

(^a^) Cases in which biopsies were performed in the middle lobe or lingula or when it was not possible to establish the exact site of the biopsy were excluded

Safety outcomes are summarized in Table 7. Pneumothorax occurred in 134 patients (19.2%), requiring chest-tube drainage in 94 cases (70.1%). The risk of pneumothorax was increased when samples were taken from different sites (*p* = 0,002), from the lower lobes (p 0,00004) and with the use of the 2.4 mm probe (*p* < 0,0001) (Table 6); it was also related with the numbers of samples (p 0,0009) (Table 5) and the lung function impairment (forced vital capacity, FVC: *p* = 0,0079; diffusing capacity of the lungs for carbon monoxide, DLCO: p = 0,0331, Table 8). Moderate haemorrhage was observed in 53 patients (7.6%) and severe haemorrhage in 5 patients (0.7%). There were no cases of fatal haemorrhage. The frequency of haemorrhage was not related to the sampling strategy (episodes of bleeding were similar if performing cryobiopsy in one site or multiple sites, both in one lobe or different lobes) (Table 6), the probe size (p 0,6460) or the severity of lung function impairment (as judged by FVC and DLCO levels). However, there was an increased risk of haemorrhage from biopsies performed in the lower lobes (p = 0,027). It was not possible to correlate the bleeding incidence with the number of samples as, after the bleeding occurred, the procedure was usually interrupted, therefore the number of samples is significantly reduced in the group of patients who developed bleeding during cryobiopsy. Incidence of moderate/severe bleeding after first sample was 15/34 (44%); moderate/severe bleeding occurring after the second sample was 13% (17/134 cases). Three patients died (0.4% of the cases): two patients died within 30 days after the procedure for acute exacerbation of IPF (the coexistence of diffuse alveolar damage and UIP was confirmed at autopsy) and one patient died two days after the procedure with thrombotic neoplastic microangiopathy/carcinomatous lymphangitis (diagnosis confirmed on histology). Characteristics of patients with more compromised lung function (FVC < 50% predicted and/or DLCO < 35% predicted) are collected in Table 9. In this specific sub-group of patients, both pathological and final multidisciplinary diagnostic yield was lower (respectively 81 and 84%), whereas there was no significant difference in terms of complications; whereas other factors related to the patient characteristics which seemed to influence the incidence of complications were the pre-test radiological pattern and pathological pattern observed in the biopsy: pneumothorax was much more frequent in patients with a higher radiological fibrotic score, evaluated grading the distribution of reticular abnormalities, traction bronchiectasis and honeycombing (p 0.04) and in patients in whom a UIP pattern was found on histology (28%, *p* < 0.0001); on the contrary, it was not possible to find any correlation between deaths or bleeding and other patients characteristics.

**Table 7:** Safety outcome

Side effects	No. (%)
Pneumothorax	134 (19.2%) - 94 drained
Bleeding	87 (12.4%) - 29 mild bleeding - 53 moderate bleeding - 5 severe bleeding
Other side effects	9 (1.3%) - 4 transient respiratory failure - 1 empyema - 1 seizures - 1 atrial fibrillation - 1 pneumomediastinum - 1 haemoptysis
Death	3 (0.4%) - 2 for AE-IPF - 1 thrombotic neoplastic microangiopathy/carcinomatous lymphangitis

Abbreviations: *AE-IPF* Acute exacerbation of idiopathic pulmonary fibrosis

**Table 8:** Correlation between safety profile and baseline lung function

	Pneumothorax	No pneumothorax	Mann-Whitney test
FVC	80.9% (41–137)	86,6% (38–143)	p 0,0079
DLCO	58,2% (25–109)	61,9% (14–129)	p 0,0331
	Bleeding	No bleeding	Mann-Whitney test
FVC	85.9% (44–128)	85.6% (38–143)	0,8909
DLCO	61.4% (26–99)	61.4% (18–129)	0,9469

Abbreviations: *FVC* Forced vital capacity, *DLCO* Diffusing capacity of the lungs for carbon monoxide

**Table 9:** Characteristic of patients with more compromised lung function (FVC < 50% predicted and/or DLCO < 35% predicted). Pre-test diagnosis was represented by NSIP (6 cases, 19%), IPF (6 cases, 19%), sarcoidosis (4 cases, 13%), diffuse neoplastic disease (4 cases, 13%), HP (3 cases, 10%), other (8 cases, 26%)

Patients characteristics (tot 31)	No. (% or SD)
Median age (SD), y	64 (7.8)
Male, No. (%)	22 (70.9)
Pathological diagnosis, No. (%)^a^	25 (80.6)
Multidisciplinary diagnosis, No. (%) ^b^	26 (83.9)
Pneumothorax, No. (%)	6 (19.4)
Drained Pneumothorax (among those with pneumothorax), No. (%)	5 (83.3)
Mild bleeding, No. (%)	2 (6.4)
Moderate bleeding, No. (%)	4 (12.9)
Severe bleeding, No. (%)	0
Other adverse events, No. (%)	1 (3.2) ^c^
2.4 mm probe, No. (%)	26 (83.9)
2 different sites, No. (%)	9 (29.0)
Number of samples (SD), No.	2.3 (1.0)

^a^Pathological diagnoses were UIP (13 cases, 42%), sarcoidosis (3 cases, 10%), adenocarcinoma (2 cases, 6%), HP (2 cases, 6.5%), OP (2 cases, 6.5%), other (3 cases, 10%).

^b^Multidisciplinary diagnoses were IPF (14 cases, 45%), sarcoidosis (3 cases, 10%), PPFE (2 cases, 6.5%), adenocarcinoma (2 cases, 6.5%), COP (2 cases, 6.5%), other (3 cases, 10%).

^c^Other adverse event: empyema; no other complications during or after the procedure

## Discussion

Indications for transbronchial lung cryobiopsy in the diagnosis of diffuse parenchymal lung diseases within the context of a multidisciplinary discussion are currently under evaluation, as well as the comparison of its risks/benefits ratio with that of surgical lung biopsy. However, reported diagnostic yields (50–100%) and observed complications of the procedure (eg, rate of pneumothorax 0–30%) vary widely in different centers [2, 5, 6] and the TLCB technique has not yet been standardized. After the rapid spread of the technique in the absence of verified competency and safety standards, in 2018 a statement by experts in the field has been published, proposing some recommendations (requisite equipment, personnel, indications/contraindications, risks and training requirements) with the aim of facilitating uniform practice and providing a guide for those wishing to introduce this technique [7]. Series reporting experience of cryobiopsy in the diagnosis of diffuse parenchymal lung diseases (DPLDs) include a limited number of patients and it is difficult to compare different series in terms of sampling strategies, procedural technical details, diagnostic yield and complications. This is the largest series of patients with suspected diffuse parenchymal lung disease who underwent transbronchial lung cryobiopsy.

In 630 cases (90.1%) lung tissue obtained from cryobiopsy, combined with clinical and radiographic information, was sufficient to establish a final multidisciplinary diagnosis for patient management. Multiple biopsies were usually taken (average number of biopsies per patient was 3.3) to reduce sampling error, as we know that diagnosis can be influenced by the heterogeneity of the disease and by the distribution of the parenchymal pathology. The optimal number of biopsies has not been established for cryobiopsy and different strategies adopted to sample lung tissue are still missing in literature. In our large series, diagnostic yield was significantly influenced by the number of samples and the sampling strategy, improving dramatically when ≥2 samples were performed (instead of only one) and when biopsy was obtained in two different sites (instead of only one site), either from the same lobe or from different lobes. This is particularly important for fibrotic lung diseases, in which pathological variability is more challenging and differential diagnosis could be more difficult; we observed discordant samples between different sites in almost 30% of cases, with a significant increase in diagnostic yield between one site and two sites. Our findings confirm and quantify the frequency of inter-lobar and intra-lobar histologic variability in fibrotic ILD and confirm the adequacy of cryobiopsy in identifying this histologic heterogeneity.

Our results confirm a previous study where biopsies from different segments within the same lobe were associated with a higher diagnostic yield compared with biopsies from the same segment [4]. In our series, cryobiopsy was always performed in different lobes in patients with significant radiographic inter-lobar heterogeneity, while in patients with diffuse radiographic pattern both in the upper and the lower lobes or in patients with a significant apical-basal gradient, cryobiopsy was more frequently performed in different segments of the same one lobe. Prior data on inter-lobar heterogeneity of DPLDs support the practice to obtain tissue from two different sites, however, histologic heterogeneity has been evaluated in the literature until now only in surgical lung biopsy (SLB) and not in cryobiopsy [8–11]. The histologic classification in 30% of the patients in our study could have differed between UIP and NSIP or UIP and HP if biopsy had been obtained in only one site; therefore, we think that it is very important to obtain tissue from two different sites, particularly when a clear apical-basal gradient cannot be identified or when different radiological patterns can be observed in different sites. Significant sampling errors may result from strategies that obtain only one biopsy specimen for ILD.

About 12.2% of cryobiopsies has been considered non-diagnostic and the reasons included inadequate alveolar tissue, normal lung tissue or minimal and nonspecific pathology. The optimal specimen size allowing pattern recognition has not been established, but some pathologists suggest that adequate specimens should measure 5 mm in diameter (which is equivalent to the size of the full field seen with a 4× objective on many microscopes) [12]. In our study, mean diameter along the shortest axis was 4.57 +/− 1.18 mm (range 0.86–9.81 mm).

Regarding complications, pneumothorax is considered the most frequent event associated with TLCB, although the rate is significantly variable in the literature, ranging from less than 1% to almost 30% [2, 5, 13–21]. In our recent meta-analysis, we have already showed that the risk of pneumothorax can be influenced by procedure-related factors, like type of sedation/airway control: a higher proportion of pneumothorax occurs among intubated patients undergoing the procedure under deep sedation with invasive jet ventilation compared to patients under sedation and spontaneous breathing [2]. In our large series, pneumothorax occurred in 19.2% of patients, requiring chest tube drainage in 70% of cases; all patients were deeply sedated and underwent the procedure intubated with a rigid tracheoscope during spontaneous breathing. We have observed that other procedure-related factors can also influence pneumothorax incidence. Pneumothorax was influenced by the number of samples and was increased when samples were taken from different sites instead of a unique site; a higher incidence of pneumothorax was associated with the use of the 2.4 mm probe compared to the 1.9 mm probe.

Bleeding during cryobiopsy can also be common [13–19, 22–25], but it is generally readily controlled endoscopically by the use of bronchial blockers and/or use of rigid tube [1, 2, 16, 23, 25, 26]. All episodes of severe bleeding reported in the literature were controlled by placement of bronchial blocker or catheter [24] and no bleeding-related deaths have been reported after cryobiopsy; a recently published report highlights the risk of potentially life-threatening complications when this precaution is not taken [27]. In our large series, we observed moderate bleeding in 7.6% of patients (requiring Fogarty balloon bronchial occlusion) and severe bleeding (resolved with prolonged Fogarty balloon bronchial occlusion, but requiring admission to intensive care unit and prolonged intubation for < 6 h) in 0.7% of patients. These results are in accordance with other papers in the literature [2, 5, 28] and confirm the importance of the use of Fogarty balloon in preventing severe bleeding. No cases of fatal bleeding were observed. Bleeding incidence was not related to the number of samples or sampling strategy (one vs multiple sites), but there was a numerical increase in the risk of bleeding if biopsy was performed in the lower lobes; one hypothesis that could explain this phenomenon is that bleeding could be caused by incidental sampling of venous vessels (however, it was not possible to discriminate between arteries and veins in the samples examination). Finally, bleeding incidence was not related with the probe size.

In our study, the mortality rate was 0.4%: two patients died within 30 days after the procedure for acute exacerbation of IPF (the coexistence of diffuse alveolar damage and UIP was confirmed at autopsy in both cases) and one patient died two days after the procedure with thrombotic neoplastic microangiopathy in the setting of carcinomatous lymphangitis. Apart from one of these 3 patients, who has been already published [1], the literature on TLCB documents 9 other deaths related with the procedure (Table 10). Mortality rate and complications associated with SLB can be influenced by comorbidities, recent disease progression [29, 30] and low baseline lung function values [31]; on the other hand, the clinical value of trans-bronchial lung cryobiopsy as a minimally invasive technique in this specific setting (patients with compromised lung function or significant co-morbidities who cannot undergo surgical procedures) has not been yet evaluated. In our large series, the risk of pneumothorax appeared increased in patients with more compromised FVC and DLCO, while bleeding was independent by baseline lung function tests. Fifteen patients had baseline FVC < 50% predicted and 22 patients had baseline DLCO < 35% predicted.

**Table 10:** Causes of death reported in literature within 30 days after transbronchial lung cryobiopsy

No	Cause of death
3	AE-IPF [1, 35] ^a^
2	Pneumothorax [35] ^b^
1	Lymphangitic carcinomatosis [23]
1	Pulmonary edema in severe aortic stenosis [36]
1	OP
1	Pulmonary embolism
1	Myocardial infarction aggravated by AE-IPF [24]

Abbreviations: *AE-IPF* Acute exacerbation of Idiopathic Pulmonary Fibrosis, *OP* Organizing pneumonia

^a^in two patients, acute exacerbation followed mechanical ventilation, necessary for respiratory failure caused by severe bleeding (no balloon or blocker used)

^b^one patient had diffuse lung adenocarcinoma and one patient had right upper lobe cavity and died from septic shock after drained iatrogenic pneumothorax.

There was no difference in terms of diagnostic yield between 2,4 and 1,9 mm outer diameter cryoprobes, although we know that in order to achieve the same specimen size, different freezing times may be necessary (5 s with the 2.4 mm probe and 7 s with the 1.9 mm probe should be sufficient in the majority of cases). However, 1,9 mm probe was associated with a significantly reduced incidence of pneumothorax compared to the 2,4 mm one and its placement in the lung periphery could be easier. Nevertheless, these results could be better confirmed by a prospective study evaluating both diagnostic yield and complications in two different randomized and homogenous groups of patients with suspected diffuse parenchymal lung diseases undergoing transbronchial lung cryobiopsy.

Regarding lung function impairment, in the restricted sub-group of patients with FVC < 50% predicted and/or DLCO < 35% predicted, both pathological and final multidisciplinary diagnostic yield was lower, whereas there was no significant difference in terms of complications; this data could be due to the fact that these patients had diseases with more complex and difficult clinical, pathological and/or radiological presentation or, most likely, to the fact that in these patients the number of samples was generally lower and samples were collected more frequently from one single site. TLCB has been performed safely in a wide age range of patients (21–87 years), with 56 patients (8%) over 75 years of age, with no complications, therefore no age limits should be suggested (giving much more importance to comorbidities and fitness for anesthesia).

This study, by nature of its retrospective design, has some limitations. 47 of 699 subjects were found to have malignancy and 38/699 sarcoidosis, the two conditions for which conventional transbronchial lung biopsy has a high diagnostic yield, however, our department is a tertiary care center and we decided to include all patients referred for suspected DPLD; this can also explain the high proportion of patients affected by CTD related ILD (50 patients, 7.2%). The availability of TLCB and its lower morbidity compared to SLB has broaden the indications for lung biopsy, including also patients with suspected occult CTD, which can be confirmed by peculiar histopathological features [14]. This study has no control group and cryobiopsy was not compared to surgical lung biopsy within the same population: SLB has never been validated as a gold standard test and side effects are not negligible [32]; for these reasons, it was not considered ethical to propose surgical biopsy to all patients independently because of the very favorable diagnostic yield obtained with cryobiopsy. Finally, diagnostic yield of cryobiopsy (both pathological and multidisciplinary) seems higher in this series compared with some previous data and other groups; these data might be overestimating the “real world” potential of TLCB and could be influenced by the expertise of the center; this procedure requires a learning curve [33] and most multidisciplinary committees do not have such degree of maximum expertise, which might easily lead to poorer results, certain reported controversy, risk-benefit concerns and clinical frustration. For this reason, it is recommended that cryobiopsy be performed by interventional pulmonologists, appropriately trained in a center with TLCB experience (familiar with advanced therapeutic bronchoscopic procedures), and be interpreted by an expert multidisciplinary team [7]. How to define cryobiopsy expertise is not an easy point and we recognize the need for training programs and the establishment of competency and quality standards for the procedure itself, in order to expand the knowledge and use of this technique, overcome the current vicious circle situation, clarify real expectations as well as the role of TLCB in clinical practice.

## Conclusion

Despite the lack of standardized procedure and approach and the heterogeneous incidence of complications in the literature, our large series confirms that lung cryobiopsy can obtain an adequate sample with a specific diagnosis in the vast majority of cases, including fibrotic lung diseases (eg. chronic HP, IPF, NSIP), with a very low overall mortality. We described some sampling strategies which seem to be associated with a higher diagnostic yield and a favorable risk/benefit ratio: 1) it is advisable to obtain two samples from two different sites in order to enhance the diagnostic yield (e.g. from different lobes in case of inter-lobar radiographic heterogeneity) and even taking samples from different segments in the same lobe may be rise the diagnostic yield, as previously demonstrated [4]; 2) it is advisable to use only 1.9 mm probe (2.4 mm probe may be associated with a higher rate of pneumothorax and more technical problems without significantly increasing the diagnostic yield); 3) sampling from lower lobes may be associated to a higher rate of complications (both bleeding and pneumothorax) than in upper lobes; 4) the risk of pneumothorax also increases in case of impaired lung function (FVC < 50% and DLCO < 35%) and sampling two sites; 5) it is preferable to intubate the patients (either with rigid tracheoscope or flexible tube), always using bronchial blockers or catheters [34]. The simplicity and low morbidity of cryobiopsy can potentially broaden the indications of this procedure compared to SLB, such as in diffuse parenchymal lung disease in patients with suspected occult collagen vascular disease, patients with more compromised baseline lung function or even in patients with a typical radiological UIP pattern, with the aim of collecting more informative data. However, low pulmonary function values have a prognostic impact and the clinical value of TLCB in this setting is not yet known; safety in the most severe group of patients would require further demonstration. Finally, the possible role and risk-benefit of TBLC in the management of patients with typical UIP pattern requires specific studies since in this group of patients, biopsy may be necessary to make a therapeutic decision only in very selected cases.

## Abbreviations

ACFE: Airway-centered fibro-elastosis
COP: Cryptogenic organizing pneumonia
CTD-ILD: Connective tissue disease – interstitial lung disease
DIP: Desquamative interstitial pneumonia
DLCO: Diffusing capacity of the lungs for carbon monoxide
DPLDs: Diffuse parenchymal lung diseases
ECD: Erdheim-Chester disease
FVC: Forced vital capacity
HP: Hypersensitivity pneumonitis
HRCT: High-resolution computed tomography
IPF: Idiopathic pulmonary fibrosis
NSIP: Non-specific interstitial pneumonia
OP: Organizing pneumonia
RB-ILD: Respiratory bronchiolitis – interstitial lung disease
SLB: Surgical lung biopsy
TLCB: Trans-bronchial lung cryobiopsy
UIP: Usual interstitial pneumonia
